# Designing Support Structures Post Sepsis in Children: Perspectives of the Queensland Paediatric Sepsis Program

**DOI:** 10.3389/fped.2021.759234

**Published:** 2021-11-18

**Authors:** Sainath Raman, Alana English, Meagan O'Keefe, Amanda Harley, Mary Steele, Jess Minogue, Kate Weller, Debbie Long, Adam Irwin, Paula Lister

**Affiliations:** ^1^Queensland Paediatric Sepsis Program, Brisbane, QLD, Australia; ^2^Paediatric Intensive Care, Queensland Children's Hospital, Brisbane, QLD, Australia; ^3^The Faculty of Medicine, Centre for Children's Health Research, The University of Queensland, Brisbane, QLD, Australia; ^4^Queensland Paediatric Rehabilitation Service, Queensland Children's Hospital, Brisbane, QLD, Australia; ^5^School of Nursing, Midwifery and Social Work, University of Queensland, Brisbane, QLD, Australia; ^6^Department of Emergency Medicine, Gold Coast University Hospital, Gold Coast, QLD, Australia; ^7^Independent Researcher, Brisbane, QLD, Australia; ^8^Centre for Healthcare Transformation, Queensland University of Technology, Brisbane, QLD, Australia; ^9^Centre for Clinical Research, The University of Queensland (UQ), Brisbane, QLD, Australia; ^10^Infectious Diseases, Queensland Children's Hospital, Brisbane, QLD, Australia; ^11^Paediatric Intensive Care, Sunshine Coast University Hospital, Birtinya, QLD, Australia

**Keywords:** sepsis, post sepsis syndrome, paediatric, consumer, family support

## Abstract

**Introduction:** Paediatric post sepsis syndrome is poorly defined and causes physical, neurocognitive, psychosocial morbidity, and family dysfunction. Families of sepsis survivors report unmet needs during care. Worldwide, the provision of post sepsis care is in its infancy with limited evidence to design clinical support pathways.

**Perspective:** The Queensland Paediatric Sepsis Program (QPSP) developed a family support structure (FSS) to improve care during all stages of childhood sepsis. It was designed in partnership with consumers guided by information from consumers and it is partly delivered by consumers. Key areas include online, multimodal education for families and the ability to connect with other families affected by sepsis. The FSS is delivered by a multidisciplinary team (MDT) acting with clinicians local to the child. Families can join the FSS registry at any stage of their sepsis journey which connects them to our MDT team and opens opportunities to participate in future research and other initiatives. Improving public awareness is a critical outcome for our consumers and they have co-designed media and digital campaigns.

**Discussion:** The ideal FSS for post sepsis syndrome management is a clinical pathway designed in partnership with consumers of interventions proven to improve outcomes from sepsis that meets their requirements. The QPSP FSS is novel as it is co-designed with, and partly delivered by, consumers with interventions aimed to improve the entire spectrum of morbidities suffered by survivors and their families, not just physical sequelae. Evaluation is embedded in the program and outcomes will guide evolution of the FSS.

## Introduction

Sepsis is life-threatening organ dysfunction due to dysregulated host response to infection (1). More than half of sepsis cases worldwide occur in children, with an estimated 25 million cases of paediatric sepsis in 2017, of whom 3.4 million died (2). Survival from sepsis has increased, but long-term morbidity experienced by paediatric survivors is increasingly apparent, both in scale and range; with effects on physical, psychosocial, educational, and family functioning (3–9). A recent International Sepsis Forum Colloquium on Sepsis Survivorship suggests “*sepsis should be viewed as a life changing and disability inducing event*” (10).

Australia was an early adopter of the 2017 WHO Sepsis resolution on sepsis, publishing “Stopping Sepsis: A National Action Plan” (11, 12). The plan has four key recommendations, including investment in support services for sepsis survivors and their families and working in partnership to design these services is emphasised: “*people recovering from sepsis and the families of patients who did not survive, remain the biggest and most important advocates for public awareness of sepsis, and it is essential they are directly involved and consulted in the design of community and peer support groups and services*” (12).

## The Queensland Paediatric Sepsis Program

A review of paediatric severe adverse events in Queensland during 2015–2016 identified sepsis as the underlying diagnosis in most cases. Contributing factors included delay in sepsis recognition and treatment, and insufficient consideration given to parental concerns (13).

The Queensland Paediatric Sepsis Program (QPSP) was established in response. It is led by a multidisciplinary team including consumers who contribute significantly to the design of the program. The aims are to reduce the burden of sepsis in Queensland and to minimise variations in sepsis care delivery.

Queensland has unique features considered in the program design; the state is geographically vast with rural communities residing distant from paediatric services and the single, quaternary children's hospital is situated in the south-east corner of the state. Additionally, Aboriginal and Torres Strait Islander children reside in greater proportion in rural Queensland and are particularly vulnerable to poor outcomes from sepsis (14).

QPSP includes interventions designed to be embedded in standard practise at every stage of sepsis. At its core, is the paediatric sepsis screening and treatment pathway. The pathway is available in all healthcare facilities, to improve quality and minimise variation in delivery of sepsis care. QPSP provides clinician education and support for quality improvement initiatives focused on early recognition, escalation and management of paediatric sepsis. A digital decision support tool is progressing for facilities with electronic health records. Additionally, the pathway incorporates antimicrobial recommendations and dashboard monitoring of antimicrobial stewardship. QPSP has a communication strategy to improve awareness of paediatric sepsis and to empower parents to seek appropriate medical assistance. Finally, the program is co-designing patient and Family Support Structures (FSS) for all phases of the disease process; the subject of this perspective.

## Reported Literature on Family Experiences and Available Support Structures

Post-sepsis syndrome was described in adults in 2012 as “new or worsening cognitive, physical, and mental health impairments that persist beyond hospitalisation” (15).

In 2019, an international colloquium on sepsis survivorship described knowledge gaps and research priorities that include characterisation of the syndrome (particularly beyond 1 year), mechanisms of post-sepsis morbidity, and the impact of interventions on outcomes (10). Data on paediatric sepsis survivorship is limited (16). The 2021 Australian Commission on Safety and Quality in Health Care literature review on sepsis survivorship (17) concluded there was a large gap in the understanding of paediatric sepsis survivorship, highlighting the need for further research.

Individual experiences of families and patients affected by sepsis are widely shared by sepsis organisations [UK sepsis Trust (18), End Sepsis (19), and Australian Sepsis Network (20)]. Common themes include delays in sepsis recognition and under-recognition of parental concerns by clinicians. While some of these organisations provide information booklets about adult post-sepsis syndrome; support structures are not described (21–23).

We present evidence in three areas relevant to paediatric sepsis: public awareness of the features of sepsis, the role of parents as experts in the assessment of their child during sepsis, the long-term effects of sepsis and the infrastructures available for children and families.

Awareness of sepsis is key to seeking early medical attention. The George Institute for Global Health surveyed the Australian public on the signs and symptoms of sepsis in 2016 and 2020 (24). In 2020, 59% of Australian adults had heard of sepsis. Younger adults, aged 18–34 years, were less likely to have heard of sepsis (45%) than adults aged over 50 years (70%). This indicates a lack of knowledge of sepsis in Australia among adults of parenting age; QPSP FSS consumer partners noted this was an area for focused improvement.

The role of parents in early detection of sepsis has not been widely examined. A recent literature review on the utility of parental concern in sepsis screening showed a single study prospectively assessing diagnostic performance (25). In this study, parental concern had high positive and negative likelihood ratios for sepsis, demonstrating a valuable contribution to sepsis recognition.

With regards to the long-term effects of sepsis in children, evidence has focussed on physical and cognitive impairments, with little focus on psychological, educational and family impacts (4–6, 8, 26). The evidence is complex as follow-up timeframes are not standardised (1–12 months) and testing parameters vary. The recent publication of a paediatric critical care core outcome set may assist in standardisation although these are not specific for sepsis (27). There are no sepsis specific support processes for children described in the literature in Australia, and only one worldwide (3). This identifies a critical knowledge gap for providing holistic family centred care.

## Needs of Families Affected by paediatric Sepsis

A pilot study in 2018 involving 19 families of children with sepsis managed in paediatric intensive care in Queensland, provided key information regarding the needs of families affected by childhood sepsis. These themes, due to be published elsewhere (MOK), have guided the development of our key family supports.

## Design and Delivery of a Paediatric Family Support Structure

The QPSP FSS is being developed and implemented collaboratively with consumers and our multidisciplinary team, led by Advanced Social Workers. The social workers have core social work skills with a person-in-environment perspective, provide interventions at a personal and social level, and specialist psychosocial knowledge and skills in sepsis care. They work with consumers, individual patients, and families, using a collaborative, strengths-based and family-centred approach and an evidence base of the grief and trauma associated with having a child with lifelong injuries due to sepsis or who are bereaved due to sepsis.

**Figure 1:**
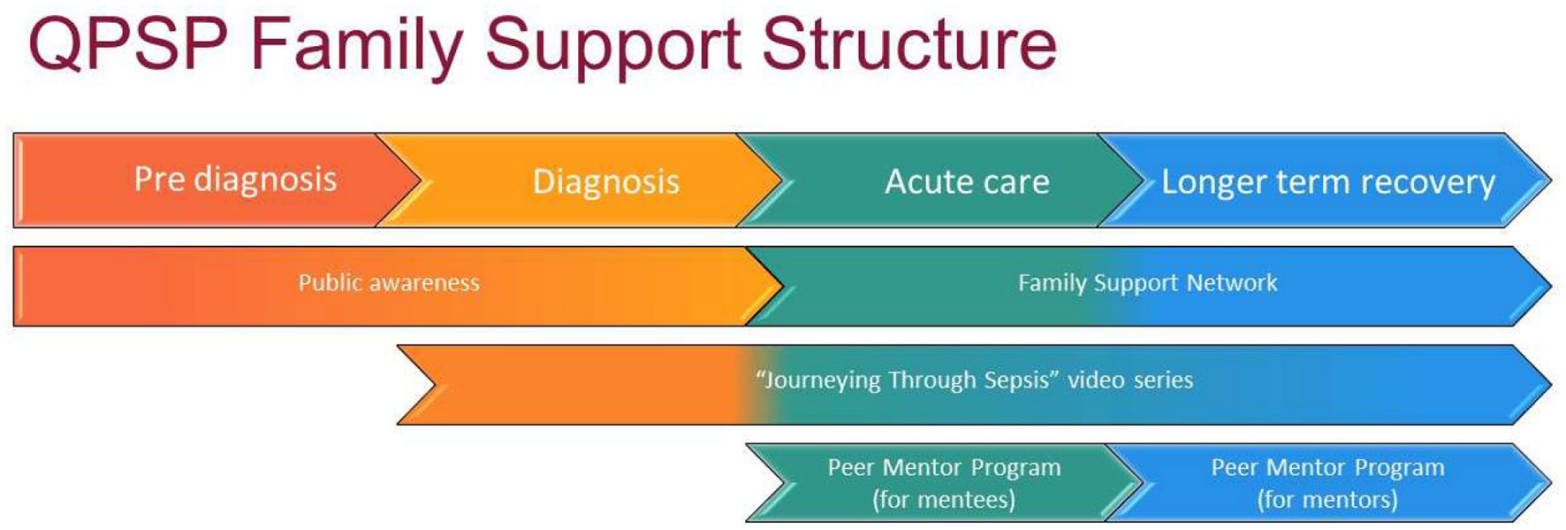
Overview of Queensland Paediatric Sepsis Program family support structure.

The FSS is an evolving model of care providing support to the child and family throughout their journey, from acute phase of diagnosis, to discharge and long-term recovery. We are engaged in an ongoing process of review and refinement of the model at this early stage of implementation. The FSS is multimodal, increasing opportunities for connection, and support for families with two priorities: (i) creating connections, psychological support, and a sense of shared experience for families through linkage with networks, peer mentor program, and direct contact with social work and (ii) education about sepsis and post-sepsis supports that is consistent and evidence-based through online videos, website links, webinars, media, and public awareness campaigns (Figure 1).

### Paediatric Sepsis Website

The Queensland Sepsis in Children Website was launched in March 2021 (https://www.childrens.health.qld.gov.au/sepsis/) (28). It responds to the need of families to access reliable information about sepsis, providing resources for families (and clinicians) and shares consumer stories to improve understanding of the experience of families affected by sepsis. There are registration links to connect to QPSP FSS and Peer Mentor Program (PMP). Website usage analytics show over 20,000-page views in the first 3 months. The most accessed materials are information for families and sepsis guidelines.

### Paediatric Sepsis FSS

We have created a registry of families through the website (28), allowing families to connect with others with similar experiences, and to be actively involved in QPSP's resource development, research and media campaigns. The registry is managed by advanced social workers who guide families towards support processes. To establish the network, we approached families already linked to the program and those referred from paediatric rehabilitation, rural and remote facilities, and Aboriginal and Torres Strait Islander community services. Clinical allied health staff across Queensland assist in promotion of the network. Registrations are increasing and include diverse representation of families across Queensland at all stages of the sepsis journey.

### Educational Video Series

An educational video series, “Journeying Through Sepsis” provides consistent information relevant to every stage of sepsis, from diagnosis through to management of post sepsis syndrome. The videos provide clinical information, validation of the family's experience, suggest support systems and strategies to facilitate resilience for the child and family. Each video is short (6–8 min) and accessible on demand, to facilitate retention of information by acutely traumatised families.

An important feature of the 8-part video series was ensuring a breadth of families could relate to the content, irrespective of location, age of child and/or level of impairment post sepsis. Four families were selected from the QPSP registry from metropolitan and rural settings, with children across the age ranges. The family's lived experience is the central message, complemented by clinicians providing consistent, evidence-based information about sepsis, its effects, and outcomes. The videos were viewed 1,400 times in the first 3 months. Promotion of the video series continues through clinical networks.

### Peer Mentor Program

Within our pilot study focus groups, families identified feeling isolated during the acute episode, in bereavement and during the daily management of the consequences of sepsis. They sought validation and normalisation of their experience and a need to connect with others who had a level of understanding of their experience. Literature suggests that peer support is a valued and unique form of assistance not typically met by the formal service systems. Mentees develop an increased sense of confidence and wellbeing, problem solving ability, adaptive coping, sense of social support and acceptance of their situation (29).

Our PMP was codesigned with consumer partners across a series of workshops, under the guidance of the Australian Centre for Social Innovation. We worked collaboratively with all key stakeholders, including St Jude's Children's Research Hospital, USA (30) to create a sustainable program with formalised recruitment, training and supervision components that ensures the safety and wellbeing of mentors and mentees.

**Figure 2:**
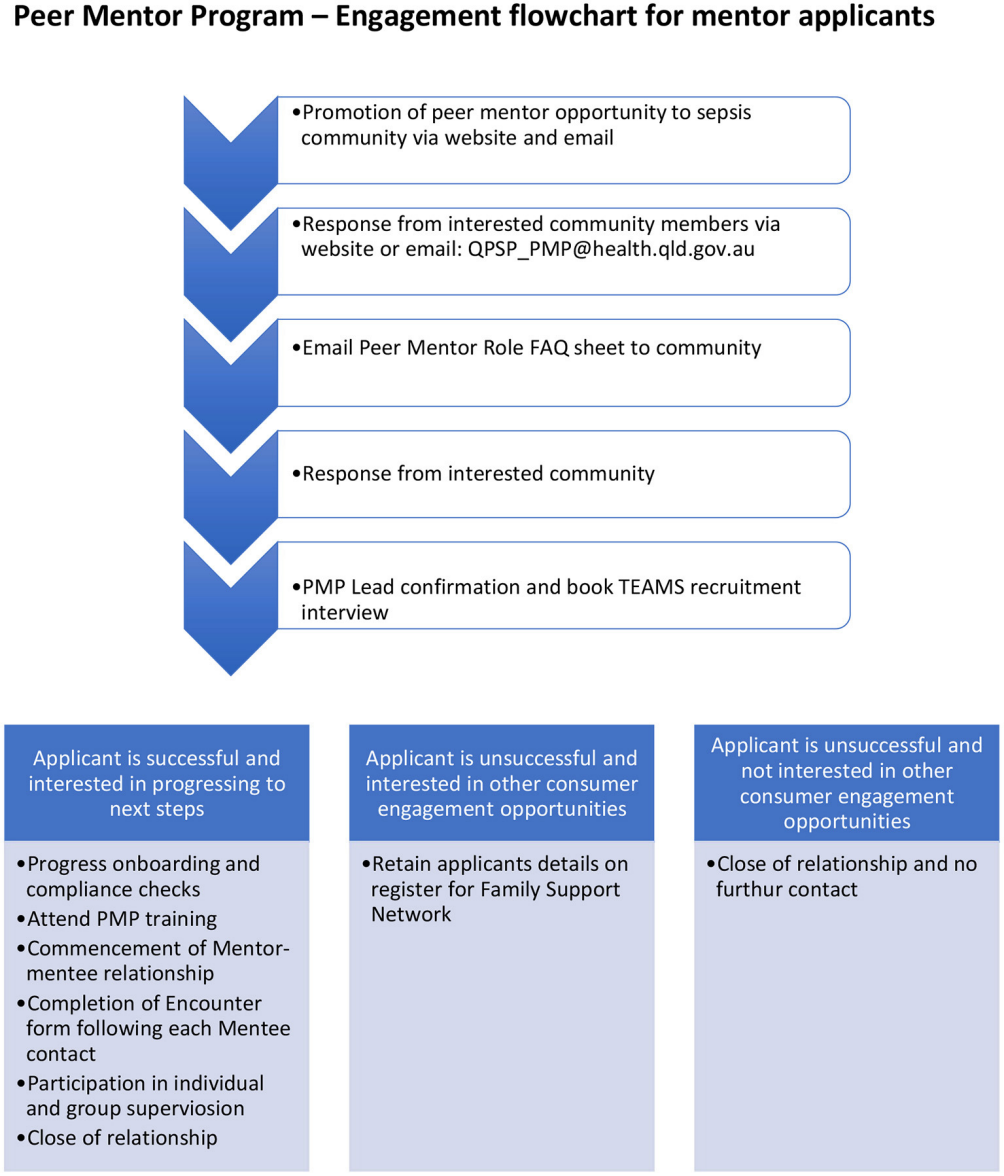
Peer mentor program engagement flowchart.

The program links families affected by sepsis with peers who have a lived experience. Parents whose child had sepsis more than 2 years previously are eligible to apply to become a Peer Mentor. Following a formal recruitment, onboarding and training process, each mentor is matched with a mentee. Peer Mentors have first-hand experience with the challenges and adjustment faced by mentees and hence can “walk alongside” the mentee, assisting them to navigate the sepsis journey. Mentors provide reassurance to mentees, normalisation of their experience, understanding of their grief and trauma and information about helpful services and resources. The PMP is available across the state on an online platform to ensure equity of access and mentors communicate using calls, video call and emails (Figure 2).

### Public Awareness Communication Strategy

Our pilot study focus group emphasised the need for improved awareness of the signs of sepsis among parents (personal communication). Digital messaging to healthcare professionals and the public is the cornerstone of our communication strategy. During a 3-month campaign in 2019, social media posts on Facebook^®^ and Instagram^®^ reached 748,341 Queenslanders and 85,531 engaged with a response or shared the content. Targeted Department of Health emails were opened by 48% of the 6,942 public recipients: with 58% clicking through to links providing further information. Sepsis emails to Queensland General Practitioners were opened by 44% of the 825 recipients: with 32% clicking through to links for further information. Digital advertisements on news and entertainment media websites linked to 2 short videos on YouTube: generating 134,251 views of the videos with a 28% completion rate. Eight media stories reached 1,003,918 readers and viewers: with an equivalent media-buy value of nearly AUD900,000. Blogs of two consumer representatives received 12,672 views. A Facebook Live series held in 2021 in partnership with CPRKids and Children's Health Queensland Hospital and Health Service had a cumulative audience of over 22,000. We note that the Queensland public had the highest awareness of sepsis (67%) amongst Australians in The George Institute Survey of public awareness in 2020 (24).

## Discussion

The ideal supports for families affected by sepsis would include interventions proven to be effective in minimising the impact of post-sepsis syndrome, that meet the needs of families from diverse backgrounds. The ideal structure covers the continuum of sepsis care, including bereavement. It should be provided by a multidisciplinary team partnered with consumers, and clinicians local to the child. However, at present, paediatric post-sepsis syndrome is neither well-described nor understood. There is limited appreciation of the nature of the syndrome, its contributing causes, and efficacious interventions. Research is hampered by poor standardisation of follow-up time periods and instruments of measurement. Sepsis can have profound effects on families (7); but there are few reports of effective interventions to reduce negative impacts. These critical knowledge gaps make the design of efficacious post-sepsis support structures difficult.

The need for support structures is acknowledged for adult survivors of sepsis and a recent review concludes that further research is required to determine the optimal approach (12, 16). In the interim, it recommends programs should employ strategies known to reduce sequalae associated with ICU- and hospital-based sepsis care. It is notable that strategies are not prioritised to enhance patient and family support and resilience, nor to promote the role of consumers in post-sepsis care or in design of the program.

The need for improved follow up for children with sepsis is highlighted by others (3). The paediatric program described by Fitzgerald benefits from consumer advocates. The group recommend multidisciplinary assessment to determine physical therapies, but psychosocial supports for the child and family are overlooked. Caregiver distress can continue for a year after the sepsis episode (7), impacting the care the family are capable of providing to the child. Family focused interventions to improve caregiver distress are important.

Examples of successful models of family support have occurred in other clinical areas, including cancer, acquired brain injury, stroke and bereavement (10, 30). The success of these models depends on consumer advocacy and funding groups partnering with research consortiums, creating momentum for evidenced-based change important to patients and families. These models have registries of patients involved in research. Consumer advocacy groups such as Global Sepsis Alliance and UK Sepsis Trust have made gains in increasing public awareness of sepsis and mobilising change, but consumers have had limited involvement in guiding research priorities (10). The international sepsis forum recommends that a global sepsis registry be established to include consumers in setting the research agenda (10).

Our FSS is novel, as it applies a multidisciplinary approach to the assessment and treatment of individual children and, in addition, it addresses the unmet needs described by families. The FSS is co-ordinated by a statewide multidisciplinary team working collaboratively with local clinicians ensuring reduced variation in care.

A core strength of the FSS is that it is co-designed by consumers and, in part, delivered by consumers; and it recognises the importance of supporting families of children with sepsis to enhance recovery. In a pilot study, families affected by sepsis described unmet needs; limited public awareness of sepsis, inadequate support, and information made available during the acute episode, limited opportunities for parental advocacy and engagement as part of the treating team and limited support after discharge (unpublished, personal communication MOK). Together with consumers, we have developed our FSS to align with the specific needs of these children and families. Co-design leads to improved satisfaction for consumers, but requires increased commitment in resourcing to partner with consumers (31).

The FFS is multimodal and multifaceted to increase opportunities for families to connect during the acute episode of sepsis or afterwards. Families access support through online registration to the Family Support Network that directs contact to the advanced social worker and creates a registry of families for the QPSP. Some consent to allow future contact regarding potential participation in QPSP initiatives, including research and related matters such as grant applications. As mentioned, the ability to connect registries of patients with future research opportunities is key to ensuring consumers can actively participate in setting the research agenda.

The multimodal FSS material ensures families access support and education in a form suited to their needs. The online platform ensures equity of access across the geographically vast state. Families from culturally and linguistically diverse backgrounds including Aboriginal and Torres Strait Islander families have differing needs and further work is being undertaken to clarify and address these in culturally appropriate ways (14). The appointment of an Aboriginal and Torres Strait Islander Lead to work collaboratively with our Social Workers is the first step in this extension of our FSS.

We implemented the FSS in two phases which has been beneficial. The first focused on primary support needs for families during the acute phase of sepsis. The second phase involves the implementation of a longer-term model of care. This addresses patient and family needs beyond discharge from hospital into the community, as ongoing impacts of sepsis become apparent (31).

As mentioned, the evidence-base for efficacy of interventions in post-sepsis syndrome is poor in children. We are in the early stages of development and implementation of our FSS, we have an evaluation plan to report on the impact of these interventions. We have embedded a Theory of Change evaluation plan within our PMP (see Supplementary Material 1). Through online evaluation tools, individual discussions and reflective practise group discussion, we aim to evaluate the impact of PMP on wellbeing and support for families affected by paediatric sepsis.

Enhancing public awareness is key to improving sepsis outcomes and an important gap identified by our consumers. The FSS campaign is based on harnessing social media platforms and organisational communication streams. The metrics detailed earlier demonstrate the engagement with our program and the ongoing work that is required in this area.

## Conclusions

Sepsis can be a “life-changing and disability-inducing event” (10). Consumers should co-design multidisciplinary structures for family support. A phased approach for FFS implementation is feasible. The first phase addresses the acute illness, promoting family education, engagement, advocacy and support. The second phase focuses on post-discharge models of care designed pragmatically in the absence of evidence. FFS implementation outcomes must be reported to allow evolution of FSS models, contributing to positive outcomes for children and families effected by paediatric sepsis.

## Data Availability Statement

The original contributions presented in the study are included in the article/Supplementary Material, further inquiries can be directed to the corresponding authors.

## Queensland Paediatric Sepsis Program

Jayde Archer, Bruce Chio, Endrias Ergetu, Kristen Gibbons, Trish Gilholm, and Nicolette Graham.

## Author Contributions

SR: conceptualisation and initial draft. DL and JM: literature review. AE and MO'K: data provision. AH, AI, KW, and MS: critical review. PL: critical review and re-writes and overall direction. All authors contributed to the article and approved the submitted version.

## Funding

The QPSP was funded by Queensland Health *via* the Care in the Right Setting project. Some of the research listed in the article was funded by a grant from the Children's Health Foundation, Brisbane Australia, awarded in 2018. Grant Reference Number 50230; total AUD99,942.

## Conflict of Interest

The authors declare that the research was conducted in the absence of any commercial or financial relationships that could be construed as a potential conflict of interest.

## Publisher's Note

All claims expressed in this article are solely those of the authors and do not necessarily represent those of their affiliated organizations, or those of the publisher, the editors and the reviewers. Any product that may be evaluated in this article, or claim that may be made by its manufacturer, is not guaranteed or endorsed by the publisher.
