# Supplementary figures and images for: Designing Support Structures Post Sepsis in Children: Perspectives of the Queensland Paediatric Sepsis Program
Source: Front Pediatr. 2021 Nov 18;9:759234. doi: 10.3389/fped.2021.759234 (PMC8636900; doi:10.3389/fped.2021.759234)

## Supplementary File:

### Proposed evaluation of PMP using Theory of Change principles

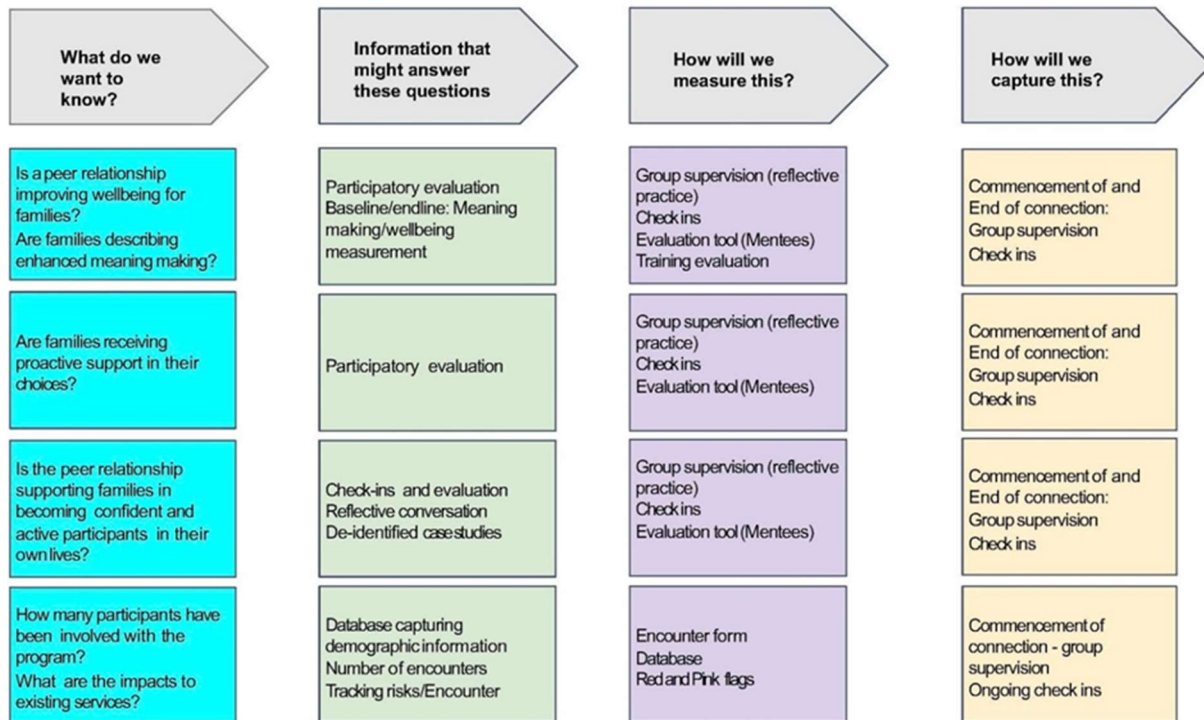

Supplement: Supplementary file 1 [file Data_Sheet_1.PDF]
